# Simultaneous parameter estimation and variable selection via the logit-normal continuous analogue of the spike-and-slab prior

**DOI:** 10.1098/rsif.2018.0572

**Published:** 2019-01-02

**Authors:** W. Thomson, S. Jabbari, A. E. Taylor, W. Arlt, D. J. Smith

**Affiliations:** 1 School of Mathematics, University of Birmingham, Birmingham, UK; 2 Institute of Microbiology and Infection, University of Birmingham, Birmingham, UK; 3 Institute of Metabolism and Systems Research, University of Birmingham, Birmingham, UK; 4 Centre for Endocrinology, Diabetes and Metabolism, Birmingham Health Partners, Birmingham B15 2TT, UK

**Keywords:** Bayesian, shrinkage, spike-and-slab, variable selection

## Abstract

We introduce a Bayesian prior distribution, the logit-normal continuous analogue of the spike-and-slab, which enables flexible parameter estimation and variable/model selection in a variety of settings. We demonstrate its use and efficacy in three case studies—a simulation study and two studies on real biological data from the fields of metabolomics and genomics. The prior allows the use of classical statistical models, which are easily interpretable and well known to applied scientists, but performs comparably to common machine learning methods in terms of generalizability to previously unseen data.

## 1. Introduction

Often in real-world regression problems, we are faced with a situation in which we have a large number of potentially irrelevant predictors, possibly even greater than the number of observations. This so-called *p* ≫ *n* problem is especially prevalent in the biological and medical sciences with the advent of high-throughput experimental methods and an increasing focus on synthesizing knowledge of molecular details into models predicting much lower-dimensional observable outcomes. Regularization and shrinkage methods aim to reduce the influence of the inherent noise in such problems and provide sparse estimated parameter vectors, essentially performing simultaneous variable selection and parameter fitting. The motivation is twofold. Firstly, regularization aims to more robustly distinguish strong from weak effects, i.e. more reliably identify the genuine driving forces of the process of interest. Secondly, we wish to reduce overfitting to improve the generalizability of our models. The performance of our method in both of these respects is demonstrated below.

The most common means of dealing with the *p* ≫ *n* problem is the LASSO [1,2], whose ability to induce genuine sparsity (i.e. estimates of *exactly* zero) and whose computationally efficient implementation make it attractive for general-purpose regularized regression. A number of Bayesian analogues of the LASSO and other penalized likelihood methods have been proposed in order to more fully account for the uncertainty in parameter estimates, which we contend is particularly important in small *n*/large *p* problems, and to tackle the tendency of the LASSO to underestimate large effects [3–5].

Some authors have focused on the subset of such problems in which the predictors have a known grouping structure, for example, in problems from genetics in which the groups correspond to known regulatory networks [6]. This has led to the development of both penalized likelihood [7] and Bayesian [8,9] modifications of common shrinkage methods.

In this paper, we present a new shrinkage prior—the logit-normal continuous analogue of the spike-and-slab (LN-CASS)—based on a logit-normal relaxation of the Bernoulli distribution used in the spike-and-slab prior [10]. The spike-and-slab is considered the gold standard of Bayesian variable selection [11], but is computationally intractable in practice due to its combinatorial complexity.

The LN-CASS prior has the advantage that its intuitive formulation allows it to be simply extended to almost any hierarchical situation—two of which are covered below—allowing the modeller to tailor the specifications of common statistical models to favour ‘simpler’ models in a variety of senses. Below we structure our models to favour first homogeneous groups of predictors before allowing within-group heterogeneity (simulation study) and to favour purely linear effects before nonlinear effects (metabolomics study), as well as applying the method in its simplest form to shrink logistic regression coefficients (microarray case study). The Bayesian formalism ‘allows the data to decide’ the appropriate level of complexity through the likelihood function.

In the simulation study, the LN-CASS prior empirically appears robust to group misspecification, and outperforms the horseshoe prior [3], the LASSO [1] and the sparse group LASSO [7]. Additionally, we apply the LN-CASS prior to a real-life classification task, in which we aim to distinguish benign from malignant adrenal tumours. Our method leads to an out-of-sample predictive performance comparable to state-of-the-art machine learning methods, but offers more interpretable results. We also use the method to build a predictive model of colon cancer malignancy using the well-known Colon dataset [12]. The code is available as a collection of r functions (see Data accessibility).

## 2. Results

To illustrate the utility of the LN-CASS prior, we conducted three comparative studies with the intention of assessing its two primary functions: identification of genuinely non-zero effects and improving out-of-sample performance by reducing overfitting. Additionally, we chose two of the three settings to highlight the flexibility of the approach, in particular its capacity to include known group structure (simulation, §2.3.1.) and to perform non-parametric regression (metabolomics, §2.3.2.). These two extensions are by no means exhaustive, but are illustrative of the myriad possible areas of application (see Discussion).

### 2.1. The logit-normal continuous analogue of the spike-and-slab prior

We now provide a brief outline of the LN-CASS prior. Mathematical details are available in electronic supplementary material, section S1.

The fundamental motivation for developing the LN-CASS prior is to provide a computationally tractable alternative to the theoretical gold standard of Bayesian variable selection, the spike-and-slab prior.

The spike-and-slab prior is based on the simple idea that, *a priori*, we believe each parameter has some non-zero probability of being zero, and the rest of the probability mass is assigned to other plausible parameter values (often uniformly). This hard zero/non-zero distinction introduces a discrete component into our prior beliefs and renders the practical use of the prior combinatorially intractable—we need to visit 2^*p*^ parameter combinations in order to adequately cover the parameter space, where *p* is the number of parameters in our model. For even moderately sized problems, this complexity renders the spike-and-slab impractical. Indeed, this combinatorial complexity is the same problem faced by the frequentist ‘best subset selection’, in which every possible subset of parameters is compared and the best performing subset is chosen.

**Figure 1.**
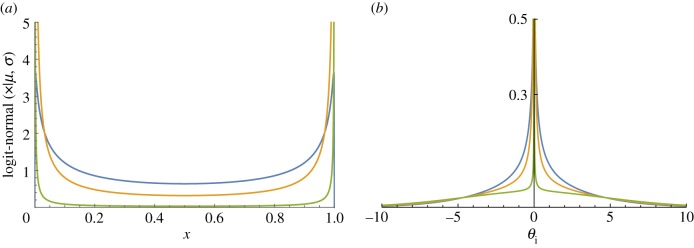
(*a*) The logit-normal distribution with *μ* = 0 and *σ* given by (blue, orange, green) = (2.5, 5, 50). (*b*) The LN-CASS priors induced by the logit-normal distributions of (*a*).

By constructing a fully continuous approximation to the mixed spike-and-slab prior, we enable greatly improved sampling efficiency at the cost of relaxing the hard distinction between zero and non-zero parameters. The LN-CASS prior accomplishes this relaxation by replacing the discrete Bernoulli distribution in the mixture formulation of the spike-and-slab with a logit-normal distribution (see the electronic supplementary material, sections S1 and S2 for details). The logit-normal distribution, with suitable parameter choices, is a U-shaped distribution on (0, 1), assigning most of its mass to values close to the endpoints (figure 1). The reason for choosing the logit-normal distribution for this purpose over the similar and more common Beta distribution is that it can be expressed as a transformation of standard normal random variables, which greatly aids the convergence properties of our sampler. Indeed, models can be specified purely in terms of parameters with (conditionally) standard normal prior distributions.

We interpret the values of the logit-normal random variable as approximate variable inclusion probabilities, which allows simple propagation of these probabilities through a hierarchical prior structure. For example, in the simulation study below we impose a hierarchical prior structure in which we favour first exclusion of whole groups of variables, then allow inclusion of groups with a shared parameter, and finally allow groups with differing parameters. In the metabolomics case study below, we use this prior structure to favour linear effects first, before allowing nonlinear effects if the data support such effects strongly enough. This corresponds to imposing a hierarchy on the complexity of the model and allows us to refine exactly how we control model complexity.

### 2.2. Performance measures

The main measure of performance we employ is the area under the receiver operating characteristic (ROC) curve (AUC). The ROC curve is a plot of the false-positive rate (specificity) against the true-positive rate (sensitivity) as the probability threshold for classifying a prediction as positive or negative is varied. The AUC is interpretable as the probability of successfully distinguishing a positive result from a negative result, i.e. the probability of correctly assigning a larger predicted value to a positive case than a negative case. An AUC of 0.5 corresponds to a model that simply uses the class proportions as a prediction, while an AUC of 1 corresponds to a classifier which perfectly distinguishes positive and negative cases at some threshold. We use the AUC to quantify the trade-off between false- and true-positives in two settings. Firstly, in the simulation study the AUC is used to quantify the degree to which each method uncovers the correct ordering of ground-truth parameter values—the degree to which genuinely small parameters are estimated to be small, and large parameters to be large. In this first case, positive and negative results are related to the sparsity pattern of the ground-truth parameter values: if a parameter has a non-zero ground truth value, it is assigned a positive result, otherwise it is assigned a negative result. The AUC, therefore, measures the probability that truly non-zero parameters are estimated to be larger (in absolute value) than truly zero parameters. Secondly, in the metabolomics and microarray case studies, we use the AUC in the more conventional setting of quantifying the out-of-sample performance of a classifier.

To quantify the agreement between the estimated and true parameters in the simulation study, we use the mean absolute error (MAE). The MAE is simply the average distance of the estimated from the true parameters.

### 2.3. Applications

We now present the results of three case studies to evaluate the comparative ability of the LN-CASS prior to perform its two main duties—sparse parameter estimation and improving out of sample performance. In the first case study, we attempt to recover ground-truth parameters in a simulation study in which we impose a known grouping structure in the predictors. In the second case study, we use real-world metabolomics data [13] to build a predictive model of adrenal tumour malignancy. In the third, we apply the LN-CASS prior in the context of Bayesian logistic regression to the well-known colon cancer dataset [12].

#### 2.3.1. Simulation study (grouped predictors)

The motivation for this case study is to illustrate the ability of the LN-CASS prior to penalize not only model complexity in terms of the number of parameters, but also the granularity of the model. Such a formulation might be applied when there is some ‘subset’ or ‘tree-like’ structure in the predictors. For example, in immunological applications, cell subsets are often nested—T-cells are subdivided into CD4^+^ and CD8^+^ T-cells, which in turn are subdivided into naive and memory subsets. The grouped LN-CASS prior favours within group homogeneity, essentially favouring less granular models, i.e. a model using total T-cell counts would be favoured over a model using CD4^+^ and CD8^+^ subsets as predictors.

We generated a simulated dataset of *n* = 100 observations from the linear regression model

1 $$y_{i}=\beta_{0}+X_{i}\beta +\varepsilon_{i},$$

for three different settings with grouped predictors, i.e. where pre-specified groups share mostly the same or similar parameter values (electronic supplementary material, table S1). The matrix **X** was sampled from a unit Latin hypercube. The *ɛ*_*i*_ were chosen to be i.i.d. zero-mean Gaussian.

We then fit the model in r with the following methods for each of the three settings (*p* = 20, 70, 120): group LN-CASS, LASSO, horseshoe, sparse group LASSO and ordinary least squares. Ordinary least squares was tested only for the *p* < *n* cases because the problem is not well defined when *p* > *n*. For code see Data accessibility. Details of the grouped LN-CASS prior are available in electronic supplementary material, section 2.1 and full details of all methods can be found in the code (see Data accessibility).

**Figure 2.**
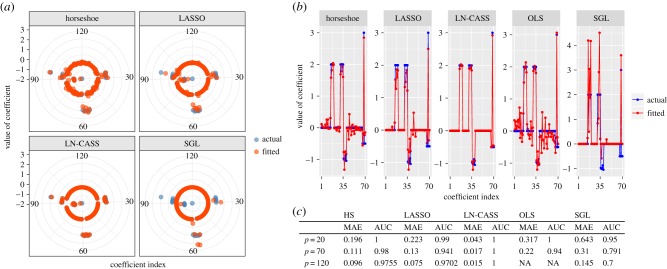
Agreement between ground truth and estimated parameters for the simulation study in the (*a*) *p* = 120 case, (*b*) *p* = 70 case; (*c*) performance measures for each method. HS, horseshoe; OLS, ordinary least squares; SGL, sparse group LASSO.

LN-CASS substantially outperforms all of the other methods in recovering the ground-truth parameters and correctly identifying zero parameters (figure 2).

#### 2.3.2. Steroid metabolomics and adrenal tumour malignancy (hierarchical GAM)

We applied a hierarchical version of the LN-CASS prior to clinical data regarding the concentrations of metabolites in the urine of patients with two different adrenal tumours. The task was to predict the tumour type based on the metabolites, and to do this we used a generalized additive model (GAM) with logit link. The implementation of the prior in a hierarchical fashion here was strongly inspired by a recent paper by Griffin & Brown [14].

The GAM we implemented represented the effect of each covariate as the sum of linear basis functions. We imposed a hierarchy through the LN-CASS prior which favoured firstly the complete removal of a covariate from the model, then inclusion of a purely linear effect, and finally allowed each of the basis functions to be used to construct a nonlinear effect (for details, see electronic supplementary material, subsection 2.2).

The dataset consisted of 158 measurements of 32 covariates [13] collected as part of the EURINE-ACT study, with 45 positive cases (malignant adrenal tumours). All of the covariates are measurements of steroid concentrations in urine samples taken from each of the patients. There is a small proportion of missing data (up to 7% of a covariate’s measurements), which we imputed via the mice() function in r [15]. We then log(1 + *x*) transformed all of the data because many of the predictors spanned several orders of magnitude. We subsequently scaled all covariates to lie in the interval [0, 1].

**Figure 3.**
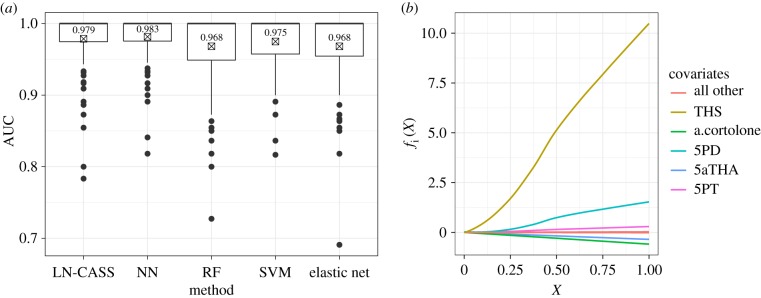
Metabolomics case study. (*a*) Boxplots of AUCs for each method computed via 16 × 10-fold cross-validation; (*b*) estimated mean functions *f*_*i*_ from the LN-CASS hierarchical GAM. Functions have been smoothed for presentation purposes with a LOESS smoother using a small span.

We compared the classification performance of our hierarchical GAM with the performance of the following methods: support vector machine (SVM), neural network (NN), random forest (RF) and elastic net (a modified version of the LASSO). Classification performance was measured using the mean AUC over 16 × 10-fold cross-validated runs. The results are presented in figure 3*a*.

All of the methods perform comparably in terms of out-of-sample predictive performance, with the NN performing the best and LN-CASS second in terms of both the mean and variability (inter-quartile range) of cross-validated AUCs. The authors are not aware of an appropriate and well-established statistical test to formalize the comparative performances of each method given the unequal variances, clear non-normality and obvious dependency between samples for a given method. However, the Kruskal–Wallis test with a *post hoc* Dunn test (and appropriate multiplicity correction) provides a non-parametric test for stochastic dominance (i.e. the tendency of values from one distribution to be larger than values from the other). We used two multiplicity corrections, both of which account for positive dependency (i.e. the tendency of large AUCs to be correlated within cross-validation folds). Using the Benjamini–Hochberg [16] correction, the only null hypotheses to be rejected at 95% significance levels were that the distribution of AUCs for the NN stochastically dominates those for the elastic net and the RF (adjusted *p*-values 0.0344 and 0.0203, respectively). Using the Benjamini–Yekutieli [17] correction, no null hypotheses were rejected; that is, no significant differences were found between the distributions in terms of stochastic dominance. Note that the Benjamini–Yekutieli correction allows for arbitrary dependencies.

The results suggest that the out-of-sample performance of the hierarchical GAM with LN-CASS prior is comparable with that of state-of-the-art machine learning methods. We argue that this performance, in conjunction with the accuracy with which LN-CASS recovers ‘true’ parameters and offers more classically interpretable results make it a valuable addition to the shrinkage and regularization toolbox for applied scientists.

The recovered effects for each of the metabolites are presented in figure 3*b*, as estimated from the full dataset. Clearly, the dominant predictor is THS which is in agreement with the original study, as are the influential roles of both 5PD and 5PT. We believe that the ability of the hierarchical GAM to produce plots such as these constitutes a considerable advantage over the machine learning methods tested and highlights the ability of LN-CASS to generate not only strong predictive models, but also to be used as an exploratory tool for the generation of hypotheses for future study.

### 2.4. Microarray data

The final case study we conducted focused on the well-known Colon dataset of Alon *et al.* [12]. The dataset consists of measurements of the expression levels of 2000 genes in 62 subjects, with the response variable being an indicator of colon cancer incidence, representing a typical *p* ≫ *n* problem in the biological/medical sciences. We compared the performance of logistic regression, with LN-CASS priors on the coefficients, to LASSO, RF and NN classifiers. We performed leave-one-out cross-validation (LOOCV) and computed the AUC across the left out samples in order to compare the estimated out-of-sample predictive accuracy of each method. In order to reduce the bias of the AUC estimates, we randomly removed an observation of the opposite class in each fold so that the class proportions were identical across folds. Code to reproduce the results of this section is available in Data accessibility and contains details of the particular implementations of all algorithms used.

We preprocessed the data by first log-transforming and subsequently standardizing (i.e. subtracting the mean and dividing by the standard deviation) the expression level of each gene. We then screened the genes via a preliminary Wald test and selected the 500 genes with the largest *Z*-scores in absolute value, leaving us with a predictor matrix consisting of the expression levels of 500 genes in 62 tissues which acted as the input to all subsequent models.

The pooled LOOCV AUCs for each method were as follows: LN-CASS, 0.904; NN, 0.8898; RF, 0.8892; LASSO, 0.858. LN-CASS performs the best, but again all of the methods perform well and there is not a substantial difference between the estimated out-of-sample performance of each method.

**Figure 4.**
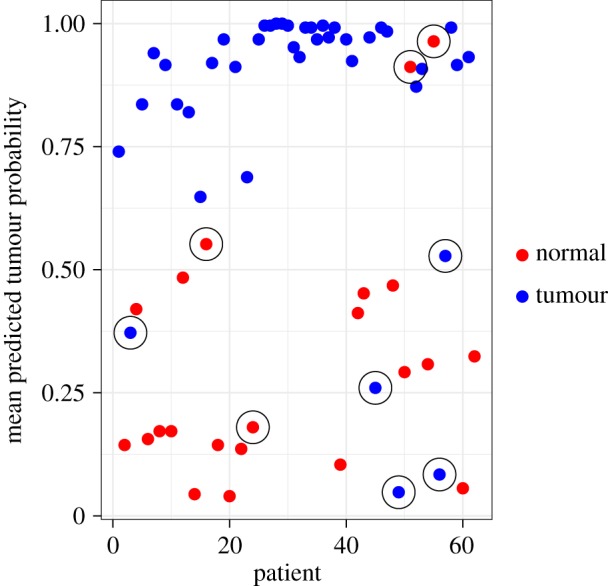
Mean predictions and observed outcomes from the LN-CASS model for the microarray data. Circled points have been identified as potentially mislabelled by [12,18].

Interestingly, there is some biological evidence for class-mislabelling, i.e. samples being incorrectly marked as either tumour or healthy [12,18] in the Colon dataset. According to Bootkrajang & Kabán [18], there are nine such samples. Figure 4 shows the mean posterior prediction for each subject with these ‘suspicious’ subjects circled. Clearly, there is reasonable agreement based on a visual inspection of the plot between the potentially mislabelled samples and those suggested by visual inspection of the LN-CASS model predictions. This suggests a possible secondary function of the LN-CASS prior in identifying mislabelled samples, the details however are left to future work.

## 3. Discussion

We have presented a new prior distribution for performing regularization/shrinkage in a Bayesian framework. We have shown that its ability to produce generalizable predictive models is comparable to state-of-the-art machine learning methods on two datasets of biological interest. Additionally, we have demonstrated with a simulation study the ability of our method to recover ground-truth parameters, even when the number of parameters is larger than the number of datapoints. In this regard, the performance of the LN-CASS prior is considerably better than other regularization/shrinkage methods which aim to estimate the parameters of classical, generative probability models (linear regression, logistic regression, etc.).

We believe that, combined, these two properties of the LN-CASS prior make it a worthwhile addition to the toolboxes of applied scientists working with typical biological datasets.

Our prior requires the choices of three hyperparameters, although we contend that they are much more interpretable than those required for other Bayesian shrinkage methods (see the electronic supplementary material, sections S1 and S2 for further detail on the roles of the hyperparameters). The three hyperparameters required correspond to, firstly, the standard deviation of the ‘slab’ component, which we refer to as *τ*; for standardized predictors, a default value of *τ* = 5 has been sufficient for all of our applications because it essentially provides a vague Gaussian prior for non-zero coefficients. Secondly, the parameters of the logit-normal distribution (figure 1*a*) must be specified; we refer to these parameters as $\mu_{\lambda }$ and $\sigma_{\lambda }$. $\mu_{\lambda }$ can be chosen based on our prior beliefs about the probability of a zero coefficient, and in our experience does not require much tuning; the median of the logit-normal distribution is given by $\text{sigm}(\mu_{\lambda })$, where sigm(⋅) is the logistic sigmoid function. Thus, if we believe *a priori* that each coefficient has a probability *a* of being non-zero, we simply set $\mu_{\lambda }=\text{logit}(a)$. $\sigma_{\lambda }$ simply controls the quality of the approximation to the spike-and-slab prior, with larger values corresponding to better approximations. We have used a default value of $\sigma_{\lambda }=10$ throughout the paper; results are not sensitive to increases in this value.

The final key advantage of the LN-CASS prior is the ease with which it generalizes to problems with a hierarchical complexity structure. This allows finer control of what exactly we mean by a ‘complex’ model, and what we mean by a desirable model—our example of using a GAM for studying the metabolomics data above illustrates this point. In that case, we imposed a hierarchical complexity structure: no effect → linear effect → nonlinear effect. In the simulation study, we favoured a complexity structure: no effect → shared group effect → individual effect. These hierarchies are accomplished simply by propagating the value of the logit-normal random variable through each layer and taking its product with a new logit-normal random variable.

We note that the prior is particularly amenable to problems in which a hierarchical complexity structure is desired, by which we mean problems in which simpler models are nested within more complex models. The simplest case is the domain of the majority of the shrinkage/regularization literature: models with fewer parameters are nested within models with more parameters. However, there are other problems with similar properties; linear models are nested within nonlinear models, models with some predictors sharing coefficients are nested within models in which each predictor has its own coefficient. One possible area of application is in multi-state survival modelling. Multi-state models describe transitions between disease states by distinct hazard functions, which may be difficult to fit with a small sample size. One might expect that the effects of many covariates remain fairly similar regardless of the state, for example age. Thus, the LN-CASS prior could be used to introduce a ‘soft’ constraint, encouraging but not enforcing covariates to share a parameter across hazard functions. This would essentially involve placing a grouped LN-CASS prior on the regression coefficients (as in the simulation study), with the groups corresponding to covariate effects.

As with most Bayesian methods, the main obstacle to the implementation of this methodology is the computational burden of MCMC sampling. Recent developments have made this procedure much more straightforward to implement and much faster [19,20]. However, for large problems this computational burden is likely to be too large to compete with the much faster frequentist and machine learning methods available. Approximate Bayesian methods offer more computationally tractable alternatives to MCMC sampling, and would be an interesting avenue of future research for this problem and allow its scalability to very large problems. In particular, non-parametric variational inference [21] appears to be the most reasonable direction, since it is able to deal both with multimodal posterior distributions and non-conjugate prior distributions.

One concern with spike-and-slab type inference procedures is the presence of multi-modal posterior distributions and the subsequent difficulty of some samplers to sample effectively from the posterior distribution, due to them becoming ‘stuck’ in local modes. We checked that the sampler we employed was able to effectively explore multi-modal posterior distributions in a linear model with interactions, a problem that is particularly prone to multi-modal posteriors. We found that whenever multi-modal posteriors appeared, they were effectively explored by multiple MCMC chains, and the Gelman–Rubin statistic [22] revealed that samples were consistent across chains. Electronic supplementary material, section S4 and the accompanying code provide more details.

The LN-CASS method does not inherently provide ‘hard’ variable selection, i.e. completely removing variables from the model, in the ilk of the LASSO. We advocate using the full model (i.e. including all predictors) for making predictions wherever possible, and using the absolute values of estimated parameters as variable importance measures for identifying the most important predictors for the purposes of hypothesis generation and/or obtaining biological insight. However, particularly in clinical/diagnostic circumstances, hard variable selection is useful to reduce the burden on clinicians/diagnosticians in collecting relevant data for using the model at the point of care.

A variety of applicable procedures for hard variable selection in Bayesian shrinkage models are available in an excellent review by Vehtari *et al.* [23]. One particularly simple method is to specify a threshold on the absolute values of the median parameters, i.e. discard all predictors whose absolute value is below some threshold. This threshold could be chosen based on the predictive performance of submodels containing only the predictors corresponding to the largest *k* coefficients in absolute value—one simply evaluates the predictive performance of each submodel and specifies a percentage of the maximum (i.e. the model including all variables) to retain.

To summarize, we have presented a flexible tool for performing regularized Bayesian regression in a variety of settings, which allows one to construct (with relative ease) problem-specific penalties on model complexity. The performance on out-of-sample data is typically at least as good as state-of-the-art methods, but the prior allows the use of classical statistical models which can be interpreted simply by applied biomedical scientists.

## Supplementary Material

Supplementary Material

## Data accessibility

Code and data used (with the exception of data for the metabolomics case study) are available on GitHub via https://github.com/willthomson1/RS-Interface-code.
