# Supplementary material for: Simultaneous parameter estimation and variable selection via the logit-normal continuous analogue of the spike-and-slab prior
Source: J R Soc Interface. 2019 Jan 2;16(150):20180572. doi: 10.1098/rsif.2018.0572 (PMC6364637; doi:10.1098/rsif.2018.0572)
Supplement: Supplementary Material [file rsif20180572supp1.pdf]

Simultaneous Parameter Estimation and Variable Selection via the LN-CASS Prior: Electronic Supplementary Material

W. Thomson[1], S. Jabbari[1,2], A. E. Taylor[3], D. J. Smith[1,3] and W. Arlt[3]

[1]School of Mathematics, University of Birmingham, UK
[2]Institute of Microbiology & Infection, University of Birmingham, UK
[3]Institute of Metabolism & Systems Research, University of Birmingham, UK

1 The Basic LN-CASS prior (Section II.C.3, Microarray Data)

The basic LN-CASS prior, as placed on the regression coefficients in the microarray case study in the main text, is used to shrink a single parameter in a non-hierarchical way, i.e. independently of the sizes of other parameters. It is the building block for the hierarchical complexity models used in the main text in the simulation and metabolomics case studies.

The marginal prior for a single regression coefficient can be specified as a scale mixture of normal densities [1] as follows,

$$\theta_i | \tau, \lambda_i \sim \mathcal{N}(0, (\lambda_i \tau)^2), \tag{1}$$

$$\lambda_i \sim \text{LogitNormal}(\mu_\lambda, \sigma_\lambda). \tag{2}$$

All experiments in the main text used fixed hyperparameters $\tau, \mu_\lambda, \sigma_\lambda$ (see also the Discussion section of main text), but of course they could be given their own hyper-prior distributions. Additionally, in the main text, the hyperparameters $\mu_\lambda, \sigma_\lambda$ are equal for each i. It is straightforward to allow different hyperparameters for each covariate to encode prior beliefs about the inclusion probabilities of the individual covariates.

The mixture parameter λ_i is analogous to an inclusion indicator for the variable of interest. Indeed, replacing the Logit-Normal prior with a Bernoulli prior yields a spike-and-slab model with a spike at 0 and a normal slab with variance τ^2, corresponding to a situation in which the ith variable is either completely excluded, or included with a $\mathcal{N}(0, \tau^2)$ prior.

Equations (1) & (2) can be rewritten in the following way, yielding a model in which inference is performed on a new parameter vector $(\boldsymbol{\theta}, \tilde{\boldsymbol{\lambda}})$ specified solely in terms of a

multivariate normal prior with diagonal covariance matrix,

$$\theta_i|\tau,\lambda_i \sim \mathcal{N}(0,(\lambda_i\tau)^2),$$
$$\tilde{\lambda}_i \sim \mathcal{N}(\mu_\lambda,\sigma_\lambda^2),$$
$$\lambda_i = \text{logit}^{-1}(\tilde{\lambda}_i).$$

This formulation, empirically, greatly enhances the performance of the No-U-Turn Markov Chain Monte Carlo sampler (NUTS) used for making posterior inference and results in much improved convergence properties over similar priors (e.g. the horseshoe).

2 Introducing Hierarchical Complexity Structure

The interpretation of the parameters λ_i as relaxed variable inclusion probabilities provides a simple way to impose soft hierarchical complexity constraints on models, by propagating these probabilities through the prior hierarchy.

The priors used for the simulation and metabolomics case studies in the main text use this structure to essentially define priors over models, with simplicity of models (in a context appropriate sense) favoured by the prior.

2.1 The grouped LN-CASS prior (Section II.C.1), Simulation Study

To wit, the grouped LN-CASS prior used in the simulation study is formulated as follows.

We assume that the coefficient for each covariate in a linear regression is composed of the sum of a group-level and a covariate-level coefficient, so that each covariate X_i is associated with a parameter

$$\beta_i = \theta_{G_i} + \theta_i$$

where G_i is the (pre-specified, perhaps by clustering) group to which covariate i belongs. Thus the effect of each covariate is due to a common effect, θ_{G_i}, among all members of its group and a deviation, θ_i, from the shared effect which is particular to that covariate.

The prior is constructed to favour exclusion of the whole group from the model, followed by inclusion of the group with a shared effect, followed by possibly distinct effects for each group element. This structure is accomplished by propagating the inclusion probabilities through the following prior structure,

$$\theta_{G_i}|\lambda_{G_i} \sim \mathcal{N}(0,(\lambda_{G_i}\tau)^2), \tag{3}$$
$$\lambda_{G_i} \sim \text{LogitNormal}(\mu_\lambda,\sigma_\lambda), \tag{4}$$
$$\theta_i|\lambda_{G_i},\lambda_i \sim \mathcal{N}(0,(\lambda_{G_i}\lambda_i\tau)^2), \tag{5}$$
$$\lambda_i \sim \text{LogitNormal}(\mu_\lambda,\sigma_\lambda),. \tag{6}$$

Equations (3), (4) specify an LN-CASS prior of the form (1)–(2) on the group level components of the parameters β_i, while equations (5), (6) specify conditional LN-CASS

priors on the individual covariate level components, with the conditioning being on the inclusion probabilities for the groups, i.e. given a small inclusion probability for the group, the individual level components are instantly assigned a small inclusion probability. However, given a group inclusion probability close to 1, the individual components are essentially assigned their own LN-CASS priors of the form (1), (2), favouring a situation in which the group share the group-level component only.

2.2 The hierarchical GAM LN-CASS prior (Section II.C.2, Metabolomics Data)

For the metabolomics case study in the main text, the LN-CASS prior was used as part of a hierarchical generalised additive model (GAM). The set up is a logistic regression problem in which we suspect that some covariates may have nonlinear effects, but we wish to let the data decide whether including such effects is worthwhile for the purposes of prediction.

Generalised additive models are extensions of generalised linear models in which the linear predictor is replaced with a sum of nonlinear covariate effects, i.e.

$$g^{-1}(y_i) = f_0 + \sum_{i=1}^{p} f_i(x_i), \tag{7}$$

with g a link function, f_0 a constant, and the f_i functions to be learnt.

We model the f_i as piecewise linear functions with some pre-specified number of knots, M. Consider, without loss of generality, the covariate space $\chi = [0,1]^p$. The functions

$$\varphi_k(x) = \begin{cases} 0, & x \leq x_k \\ \frac{x-x_k}{1-x_k}, & x > x_k \end{cases}, \; x \in [0,1], \; k \in 1, \ldots, M, \tag{8}$$

form a basis for the space of piecewise linear functions with M knots on $[0,1]$, which are 0 at the origin. We therefore represent each f_i as a linear combination of these basis functions,

$$f_i(x_i) = \sum_{k=1}^{M} \omega_{k,i}\varphi_k(x_i). \tag{9}$$

The weights ω_k are the subject of the hierarchical complexity shrinkage procedure, and the structure is very similar to that employed by the grouped LN-CASS prior. The motivation is that, if the ω_{k_i} are all 0 for a given, i, the covariate has no effect, if we allow $\omega_{1,i}$ to be non-zero, we obtain a linear effect, and if other weights are allowed to be non-zero we obtain a piecewise linear effect. This is the complexity hierarchy we wish to impose.

Thus, the prior on the weights is as follows

$$\omega_{1,i}|\lambda_{1,i} \sim \mathcal{N}(0, (\lambda_{1,i}\tau)^2), \tag{10}$$
$$\lambda_{1,i} \sim \text{LogitNormal}(\mu_\lambda, \sigma_\lambda), \tag{11}$$
$$\omega_{k,i}|\lambda_{1,i}, \lambda_{k,i} \sim \mathcal{N}(0, (\lambda_{1,i}\lambda_{k,i}\tau)^2), \text{ for each } k = 2, \ldots, M \tag{12}$$
$$\lambda_{k,i} \sim \text{LogitNormal}(\mu_\lambda, \sigma_\lambda), \text{ for each } k = 2, \ldots, M. \tag{13}$$

	Zero groups	Constant groups	Noisy groups	Disparate groups
$p = 20$	$\beta_{G_1}, \beta_{G_3} = \mathbf{0}$	$\beta_{G_2} = \mathbf{2}$	$\beta_{G_4} = -\mathbf{1} + \mathcal{N}(0, 0.1^2)$	
$p = 70$	10 zero groups total.	$\beta_{G_4}, \beta_{G_7} = \mathbf{2}$	$\beta_{G_8} = -\mathbf{1} + \mathcal{N}(0, 0.1^2)$	$\beta_{G_{14}} = (-0.5, -0.5, 3, -0.5, -0.5)$
$p = 120$	20 zero groups total.	$\beta_{G_6} = \mathbf{1}$	$\beta_{G_{12}} = \mathbf{2} + \mathcal{N}(0, 0.1^2)$	$\beta_{G_{18}} = (-1, -1, -1, -2, -1)$<br>$\beta_{G_{19}} = (0.5, 0.5, 0.5, 2, 0.5)$

Table 1: True coefficients for each of the simulation experiments. Bold numbers represent constant vectors of length 5 and we abuse the $\mathcal{N}(0, 0.1^2)$ notation to mean a vector of 5 samples from a normal random number generator with mean 0 and standard deviation 0.1.

Again, by rewriting this in terms of logit transformed normal random variables, we obtain a multivariate normal prior on our parameters of interest.

3 Simulation study details

Details of the method for generating the synthetic data used in the simulation study (Section II.C.1) are presented here. Table 1 outlines the ground truth parameters used to generate the data. We drew 100 synthetic covariate vectors from a unit latin hypercube and simulated data from a linear regression with additive Gaussian noise using the parameters generated by the procedure outlined in Table 1.

4 MCMC mixing and multi-modal posteriors

Multi-modal posterior distributions are a common complication of spike-and-slab type prior distributions. Most often when multi-modality occurs, the marginal posteriors for some parameters have a mode centred at zero and a second mode elsewhere. This multimodality is particularly prevalent in, for example, linear models with interactions. Random-walk based samplers such as Metropolis-Hastings or Gibbs samplers can experience difficulties effectively exploring multi-modal posteriors. It is often claimed that the No U-Turn Sampler deployed by Stan is much better at exploring multimodal posterior distributions than other common MCMC samplers.

We verified that this was indeed the case in our application with a simulated dataset and a linear model with interactions. See the Data Accessibility section online for access to the code, which also provides full details on the methodology.

We used a simple visual check to examine the mixing of the sampler. We ran four randomly initialised chains for 4000 iterations each, discarding the first 2000 samples as burn-in. Subsequently, we examined kernel density estimates of the marginal posteriors for each parameter and each chain. Whenever there appeared to be a bimodal marginal posterior distribution in any of the chains we checked that the other chains had also explored both modes. This was always the case, suggesting that the sampler is able to consistently and robustly escape local modes. Figure 5 shows trace plots and kernel density estimates for each chain of a representative multi-modal marginal posterior distribution. The full posterior can be explored using the available code. Figure 6 shows a histogram of the Gelman-Rubin statistics for the parameters subject to

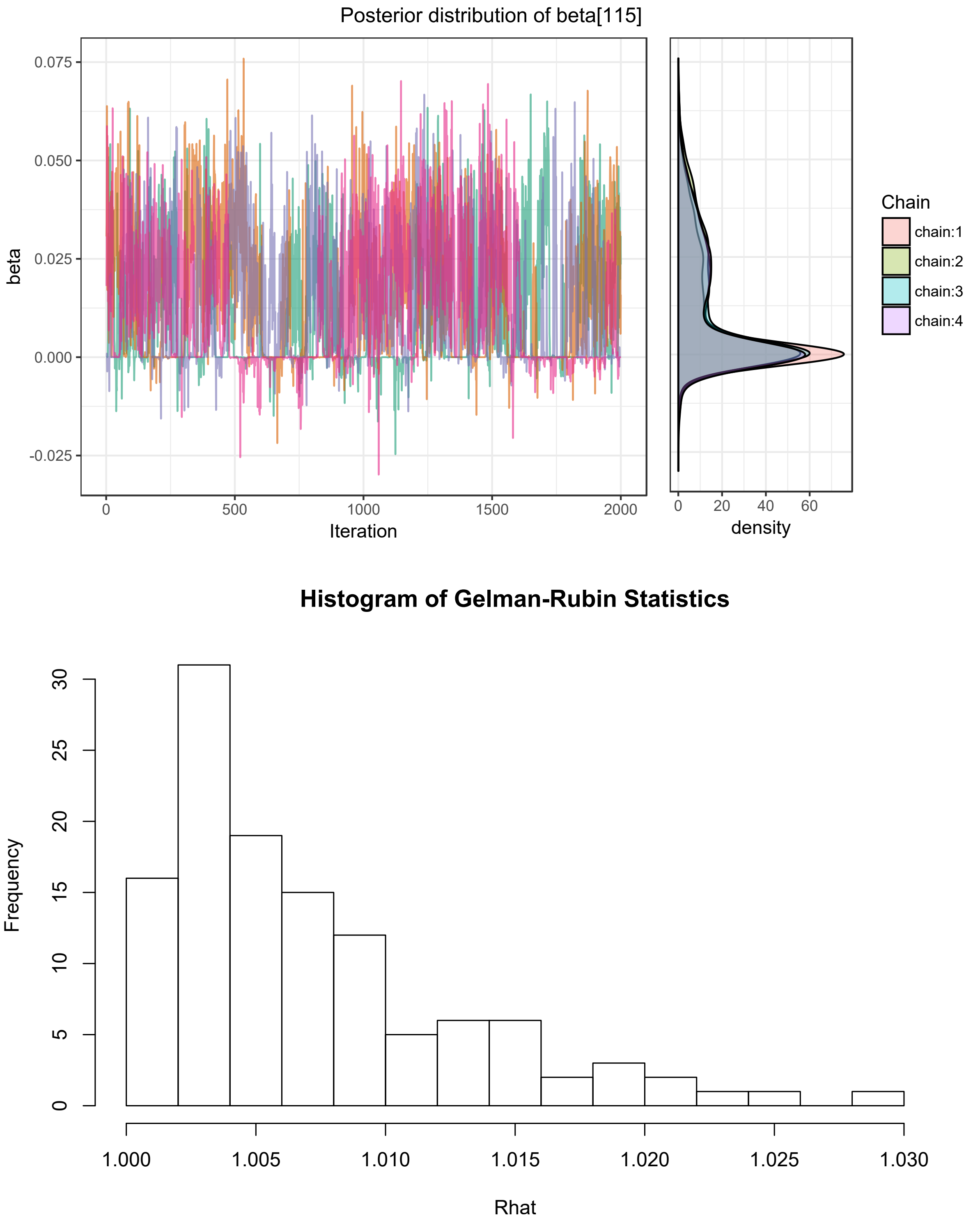

Figure 1: Top: Trace plots and kernel density estimates for one of the parameters with a multi-modal posterior distribution for four separate chains. Bottom: Histogram of the Gelman-Rubin statistic $\hat{R}$ for the parameters subject to the LN-CASS prior.

LN-CASS priors. They are all close to one, suggesting that mixing is sufficient.

References

[1] Andrews, D.F. & Mallows, C.L., Journal of the Royal Statistical Society. Series B (Methodological) **36** (1974)
